# Self-Assembly of the Block Copolymer Containing Discotic Mesogens Driven by Liquid Crystalline Ordering Effect

**DOI:** 10.3390/polym16233339

**Published:** 2024-11-28

**Authors:** Xiaojian Hou, Lingjuan Hu, Huanzhi Yang, Bixin Jin, Yunjun Luo, Xiaoyu Li

**Affiliations:** 1 School of Materials Science and Engineering, Key Laboratory of High Energy Density Materials (MOE), Beijing Institute of Technology, Beijing 100081, China; 3120205579@bit.edu.cn (X.H.); 3120205580@bit.edu.cn (L.H.); 3120195592@bit.edu.cn (H.Y.); yjluo@bit.edu.cn (Y.L.); 2 Zhuhai Campus, Beijing Institute of Technology, No. 6 Jinfeng Road, Tangjiawan, Xiangzhou District, Zhuhai 519000, China

**Keywords:** self-assembly, liquid crystalline block copolymer, liquid crystalline ordering effect, discotic mesogen

## Abstract

Block copolymers (BCPs) have attracted considerable attention due to their ability to form a variety of complex assemblies with diverse morphologies and functions in solution. By incorporating liquid crystalline (LC) moieties, the LC side chains significantly affect the morphologies and sizes of BCP assemblies. In this study, we synthesized the copolymer with an LC block containing triphenylene (HAT) discotic mesogen and short methylene side chains. By enhancing the π–π interaction between triphenylene discotic mesogens, and doping the discotic mesogens, the LC orderedness was significantly enhanced and able to dictate the self-assembly behaviors of the BCP in solution. Additionally, the lengths of resultant fibrillar micelles were easily tuned by adjusting the dopant content. More interestingly, two growth modes, nucleation growth and coupling, were observed during the formation of fibrils. Consequently, with long-term aging and sufficient concentration, a large portion of these fibrils underwent end-to-end coupling to form long fibrils, allowing the formation of organogel via inter-fibrillar entanglement.

## 1. Introduction

During the past decade, the solution-state self-assembly of block copolymers (BCPs) has drawn tremendous research attention as one of the most efficient approaches to fabricating nanoassemblies of various morphologies [1,2,3,4,5]. Many methods have been developed to control the morphologies of micelles, such as adjusting the solvation environment [6,7,8,9,10], adding small molecules [11,12,13], changing the block ratio of BCPs [14,15,16], and introducing crystalline [17,18,19,20] or LC blocks [21,22,23,24]. Especially for LC BCPs, the formation of the LC phase within the micellar core can lead to more diverse morphologies [13,25]. In recent years, micellar nanostructures with different morphologies (such as sphere, cylinder, vesicle, etc.) have shown great potential applications in various fields, such as drug delivery [2,26,27,28], nanoreactors [29,30], optoelectronic devices [31,32], nanocatalysis [33], and stimuli-responsive materials [34,35].

Side-chain LC BCPs can be divided based on the shapes of the mesogens, such as rod-like mesogens [22,36,37,38], discotic mesogens [39,40,41,42], and dendronized LC BCPs [43]. When rod-like mesogenic motifs are incorporated into the side chain, they can self-assemble into nematic [44], smectic [45], and cholesteric phases [46]. Different from rod-like mesogens, discotic mesogens can organize into a column via π–π interaction and then self-assemble into columnar phases [47,48]. Although discotic LC polymers combine directional molecular stacking and fluidic properties and have been found to be attractive for thermal [49], optical [50,51,52,53], and electronic devices [54], their solution-state self-assembly behavior has been rarely explored, especially for copolymers with an LC block containing triphenylene (HAT) discotic mesogen. Moreover, HAT4 is one of the most typical discotic molecules, with a symmetric triphenylene planar core and butyloxy side chains around the disk. The LC orderedness of the discotic LC BCPs can be regulated by the property and grafting density of LC mesogens, structure and length of flexible spacers, lengths of tails, etc. [55]. Among these factors, altering the tail length can effectively influence the assembly structure of discotic LC BCPs. However, most of the studies on the self-assembly behavior of side-chain LC BCPs containing discotic mesogen mainly focus on the bulk system. The self-assembly of LC BCPs containing HAT discotic mesogen in selective solvents has barely been explored.

In our previous work, the unusually convoluted self-assembly behaviors of an LC BCP containing HAT discotic mesogens and aliphatic hexyl tail chains were investigated [56]. The micelles underwent multiple morphological transitions spontaneously, driven by their intrinsic subtle LC ordering effect. Moreover, the introduction of electron donor 2,4,7-trinitrofluorenone (TNF) significantly accelerated the morphological transition process by forming electron donor–accept complexes and building LC orderedness very quickly. The LC ordering effect was so weak that the mesophase was gradually built inside the micellar core and changed the micellar morphologies.

**Figure 1:**
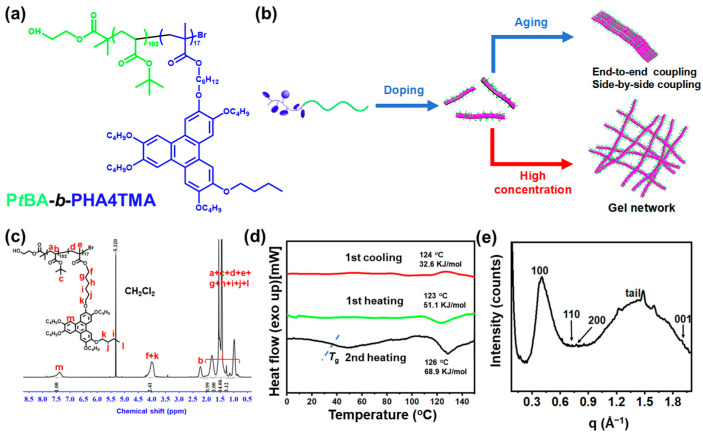
(**a**) Chemical structure of P*t*BA_102_-*b*-PHAT4MA_17_, (**b**) Schematic illustrations of the fibril and gel network formation process driven by the mesogenic ordering effect, (**c**) ^1^H NMR spectrum of P*t*BA_102_-*b*-PHAT4MA_17_, (**d**) DSC trace (blue dashed line refers to *T*_g_ of the BCP) and (**e**) WAXS spectrum of the bulk P*t*BA_102_-*b*-PHAT4MA_17_.

In this work, we synthesized an LC BCP poly(*tert*-butyl acrylate)-*block*-poly(6-(3,6,7,10,11-pentakis(butyloxy)-2-oxytriphenylene) hexyl methacrylate) (P*t*BA_102_-*b*-PHAT4MA_17_, Figure 1a) containing a butyl side chain and studied the self-assembly behaviors of the LC BCP in solution driven by the LC ordering effect. Compared with the previous work, the shorter flexible chain reduced interference in the π–π interaction between HAT discotic mesogens and significantly enhanced the LC orderedness. The influence of LC ordering effect on the assembly behavior of the BCPs in solution was investigated in detail. It was found that when TNF was introduced to dope the mesogens, the orderedness would also be further enhanced to produce fibrils with different lengths very rapidly. Two growth modes, nucleation growth and coupling, were observed during the formation of fibrils. Consequently, very long fibrils were obtained after being aged for a long period or sufficient concentration, resulting in the formation of organogels via inter-fibrillar entanglement at a decent concentration (Figure 1b).

## 2. Materials and Methods

### 2.1. Materials

Resorcinol, *tert-*butyl acrylate (*t*BA, 99%), and 1,2-dibutoxybenzene were purchased from the Tokyo Chemical Industry (TCI, Tokyo, Japan). Catechol (99%), potassium carbonate (K_2_CO_3_, 99%), methanol (MeOH, 99%), 2-propanol (2-PrOH, 99%), ethanol (EtOH, 99%), dichloromethane (CH_2_Cl_2_, 99%), tetrahydrofuran (THF, 99%), cuprous bromide (CuBr, 99%), *N*, *N*, *N*′, *N*″, *N*″-pentamethyl diethylenetriamine (PMDETA, 99%), calcium hydride (CaH_2_, 99%) and catechol (99%) were all purchased from Sigma-Aldrich (St. Louis, MO, USA). Methanol (MeOH), ethanol (EtOH), dichloromethane (CH_2_Cl_2_), and tetrahydrofuran (THF) were distilled over CaH_2_ before use. CuBr was purified with acetic acid before use. All other chemicals were used as received without further purification.

### 2.2. Methods

#### 2.2.1. Transmission Electron Microscopy (TEM)

The samples for TEM were produced by drop-casting approximately 5 μL of the solution onto a copper grid and placed on a piece of filter paper to quickly remove the excess solvent in 1 s to prevent further morphological change. The grid was captured with bright-field TEM from the Japanese company JEOL (Tokyo, Japan) at an acceleration voltage of 80 kV. The images were analyzed using the Image-Pro Plus 6.0 software. For the statistical length analysis, a minimum of 300 micelles were carefully traced manually to determine their lengths. The number average micelle length (*L*_n_) and weight average micelle length (*L*_w_) were calculated from measurements of the contour lengths (*L*_i_) of individual micelles, where *N*_i_ is the number of micelles of length *L*_i_, and *n* is the number of micelles examined in each sample. The distribution of micelle lengths (PDI) is characterized by *L*_w_/*L*_n_.

(1) $$L_{n}=\frac{\sum_{i=1}^{n}N_{i}L_{i}}{\sum_{i=1}^{n}N_{i}}$$

(2) $$L_{w}=\frac{\sum_{i=1}^{n}N_{i}L_{i}^{2}}{\sum_{i=1}^{n}N_{i}L_{i}}$$

#### 2.2.2. Gel Permeation Chromatography (GPC)

The molecular weight and polydispersity index (*M*_w_/*M*_n_) of polymers were obtained by gel permeation chromatography (GPC, Shimadzu Corporation, Kyoto, Japan) using a Viscotex GPC max Chromatograph equipped with styrene/divinylbenzene columns. Butylated hydroxytoluene (0.025 wt.%)-stabilized THF was used as the eluent, with a flow rate of 1.0 mL/min. Samples were dissolved in the eluent (10 mg/mL) and filtered (Acrodisc, PTFE membrane, 0.22 μm) before injection. The calibration of the refractive index detector was carried out using polystyrene standards (Viscotek).

#### 2.2.3. Nuclear Magnetic Resonance (NMR)

^1^H NMR and ^13^C NMR spectra were produced by the Avance 500 from Brucker (Billerica, MA, USA; operating at 500 MHz). The solvents used were CDCl_3_ and CD_2_Cl_2_, with tetramethylsilane (TMS) as the internal standard. The sample was tested at a concentration of 5 mg/mL.

#### 2.2.4. Differential Scanning Calorimetry (DSC)

The diblock copolymer samples used for the DSC (Bruker, Billerica, MA, USA) experiment were obtained by annealing the sample at 35 °C for 24 h. The fibril samples used for the DSC experiment were dry powders obtained by drying the original fibril solution in a vacuum oven at 40 °C for 24 h. The heating and cooling rate was 10 K/min, and the test temperatures ranged from −30 to 200 °C. The sample mass of 5 mg was placed in an aluminum crucible.

#### 2.2.5. Wide-Angle X-Ray Scattering (WAXS)

The scattering patterns were recorded with the Ganesha system (SAXSLAB, Amherst, MA, USA) equipped with multilayer focused Cu Kα radiation as the X-ray source (Genix3D Cu ULD) and a semiconductor detector (Pilatus 300 K, DECTRIS, Baden, Switzerland).

#### 2.2.6. Atomic Force Microscopy (AFM)

AFM experiments were conducted directly on the carbon-coated copper grid used for TEM analysis. AFM images were recorded by using a Dimension FastScan (Bruker, Billerica, MA, USA). The images of the fibrils were acquired in tapping mode in the ambient environment.

#### 2.2.7. Synthesis of P*t*BA_102_-*b*-PHAT4MA_17_

In a dry Schlenk tube, 154.6 mg (0.2 mmol) of 2-(6-methacryloxyhexoxy)-3,6,7,10,11-pentabutoxybenzothiazole, 260 mg (0.02 mmol) of the macromolecular initiator P*t*BA_102_, 8.4 μL (0.04 mmol) of PMDETA, and 2 mL of 1,4-dioxane were sequentially added. The mixture was stirred magnetically until well mixed and dissolved. After two freeze–pump–thaw cycles, 5.8 mg (0.04 mmol) of copper(I) bromide was quickly added to the Schlenk tube, followed by another freeze–pump–thaw cycle. The Schlenk tube was placed in an 80 °C oil bath for 6 h, and then the reaction was quenched with liquid nitrogen.

THF (10 mL) was added to dilute the polymer solution. The catalyst was removed by passing the solution through a neutral alumina column, and the residual monomers were separated via silica gel column chromatography. The polymer was eluted with ethyl acetate, and the concentrated crude product was further purified by repeated precipitation from the THF solution into MeOH three times. The precipitate was collected via centrifugation and vacuum-dried at room temperature to yield 181.5 mg of white polymer (yield = 41%).

Details for the synthesis and characterization of other molecules and polymers are recorded in the Supplementary Materials (Figures S1–S10).

## 3. Results and Discussion

The LC BCP was synthesized via sequential atom transfer radical polymerization (ATRP), as illustrated in Figure S11. The ^1^H NMR spectrum in Figure 1c shows that the chemical shifts of the double-bond protons of HAT4MA at 6.10 ppm and 5.54–5.53 ppm disappeared, indicating the LC BCP was synthesized successfully. The degree of polymerization (DP) of PHAT4MA was calculated to be 17. The molecular weight distribution was determined with gel permeation chromatography (GPC, Figure S12, Table S1). The GPC curve for P*t*BA_102_-*b*-PHAT4MA_17_ revealed a single peak with a number average molecular weight (*M*_n_) of 21.1 kDa and a low polydispersity index (PDI = 1.24).

The LC phase structure of HAT mesogens has been well understood. In the current research, the mesophase to isotrope phase transition of HAT mesogens was determined with differential scanning calorimetry (DSC) to be 126 °C, with an enthalpy value of 68.9 KJ/mol (Figure 1d), which would increase to 71.22 KJ/mol if it was pre-annealed at 35 °C for 24 h (Figure S13). In the previous example, the polymer containing HAT with hexyl side chains needed a pre-annealing to show a decent phase transition around 43 °C with an enthalpy of 63.4 KJ/mol [56]. This finding suggests that the shorter alkyl side chains of such discotic mesogen-containing LC BCP significantly enhanced the LC ordering effect of the polymer.

The wide-angle X-ray scattering (WAXS) spectrum of P*t*BA_102_-*b*-PHAT4MA_17_ was obtained to reveal the molecular packing of the HAT mesogens. As shown in Figure 1e, characteristic diffraction peaks corresponding to (100), (110), and (200) planes were visible, indicating the presence of a hexagonal columnar LC phase (Colh). Based on the equations

(3a) $$d=\frac{2\pi }{q}$$

(3b) $$\frac{1}{d_{hk}}=\frac{4(h^{2}+k^{2}+hk)}{{3_{a}}^{2}}$$

the hexagonal lattice parameter is calculated to be *a* = 17.84 Å^−1^. The diffraction peak in the wide-angle region at *q* = 1.901 Å^−1^ (*d* = 3.31 Å) corresponds to the crystalline plane (001) from the π–π stacking interaction. The diffraction peak at *q* = 1.35 Å^−1^ (*d* = 4.65 Å) represents the disordered arrangement of alkyl chains. The closer packing of HAT discotic mesogens in the PHAT4MA block than their counterparts with hexyl side chains (3.53 Å) confirms their higher LC orderedness.

The LC behaviors of the BCP were also observed via polarized light optical microscopy (POM). This sample was loaded onto the fused quartz plate pre-heated at 150 °C to erase the thermal history of the BCP, and then cooled to room temperature. As shown in Figure S14, the sample exhibited distinct birefringent structures at room temperature, indicating that the PHAT4MA block formed a mesophase. As the temperature increased to 126 °C, the sample still showed birefringent structures. However, when the temperature reached 128 °C, the birefringent structure became less pronounced; and at 130 °C, no birefringence was detected, indicating that the sample was in an isotropic state. This suggests that the liquid crystal transition temperature is around 130 °C, which is consistent with the DSC results.

**Figure 2:**
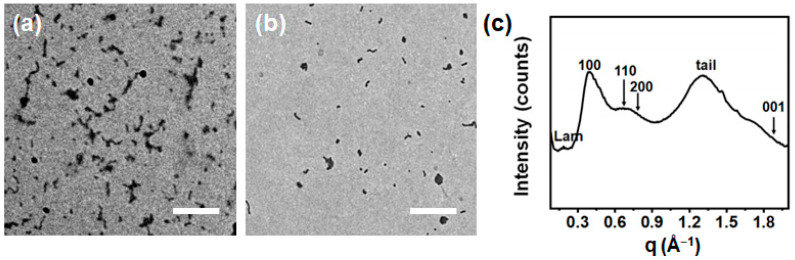
TEM images of the self-assembled structures produced by dispersing the P*t*BA_102_-*b*-PHAT4MA_17_ (0.1 mg/mL) in (**a**) 2-PrOH and (**b**) ethanol at 80 °C for 1 h and cooling down to r.t. naturally. Scale bars are 2 µm. (**c**) WAXS spectrum of the P*t*BA_102_-*b*-PHAT4MA_17_ rod sample dried from its 2-PrOH solution.

In self-assembly experiments, the LC BCP was thermally dispersed (80 °C for 1 h) in 2-PrOH or EtOH (0.1 mg/mL) and then cooled naturally to room temperature (r.t., 21 °C) within 3 h without stirring. The samples were drop-cast onto the carbon-coated copper grid and observed under the transmission electron microscope (TEM). As shown in Figure 2a,b, very short fibrils were observed in both solvents. The block ratio was calculated to be 0.99:1.00 (corona:core, w.t.). Without the formation of LC phase in the core, this diblock copolymer would tend to form vesicular micelles in selective solvents, based on the classic theory for coil–coil diblock copolymers.

Therefore, the mesogenic packings inside the micellar core were also characterized with WAXS. The discotic mesogens formed a similar Colh phase with a hexagonal lattice parameter (*d* = 18.85 Å) in the micellar core (Figure 2c). Diffraction peaks were observed at *q* = 0.385 Å^−1^ (*d* = 16.32 Å), *q* = 0.666 Å^−1^ (*d* = 9.43 Å), and *q* = 0.78 Å^−1^ (*d* = 8.06 Å), corresponding to the (100), (110), and (200) planes, respectively. Another diffraction peak at *q* = 1.87 Å^−1^ (*d* = 3.36 Å) corresponding to the (001) plane was also observed, representing π–π stacking, and a diffraction peak at *q* = 1.315 Å^−1^ (*d* = 4.78 Å) represented the disordered stacking of the alkyl chains. Moreover, the diffraction peak at *q* = 0.187 Å^−1^ (*d* = 33.60 Å) in the small angle region was twice the *q*-value of the main diffraction peak (100) plane, confirming the formation of additional lamellar phase structure inside the LC fibrillar core. The mesogenic packings of the HAT motifs in the bulk and the micellar core were very close. This finding suggests that the mesogenic ordering effect of PHAT4MA was sufficiently strong that the LC mesophase was built very quickly during the micellization process, instead of slowly after the micellization when hexyl side chains for HAT discotic mesogens were adopted [56].

To further enhance the LC ordering effect, small molecule receptors (such as 2,4,7-trinitro-9H-fluoreno-9-ketone, TNF) were added into the system to dope the HAT mesogens by forming an electron donor/acceptor (EDA) complex via charge transfer (CT) [57]. The doping ratio (*r*) is defined as the mole ratio of electron acceptor (TNF) to HAT motifs in the LC BCP. TNF dopants were added to the THF solution of the LC BCP to allow the full mixing and the formation of EDA complexes between TNF and HAT moieties at a concentration of 10 mg/mL. The solvent was then evaporated and the complexed BCPs were thermally dispersed into 2-PrOH (80 °C, 1 h) and cooled down naturally to r.t. within 3 h.

**Figure 3:**
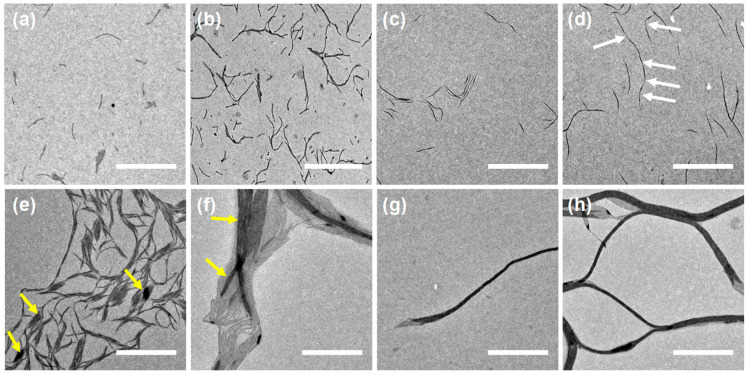
TEM images of the self-assembled structures produced by dispersing the doped P*t*BA_102_-*b*-PHAT4MA_17_ (0.1 mg/mL) with *r* = (**a**) 0.1, (**b**) 0.2, (**c**) 0.3, (**d**) 0.4, (**e**) 0.5, (**f**) 0.7, (**g**) 0.8 and (**h**) 1.0, in 2-PrOH at 80 °C for 1 h and cooling down to r.t. naturally. The white arrows were indicated to the fibrils end-to-end coupling connection. The yellow arrows were indicated to the formation of nanoribbon-like structures through side-by-side coupling of the individual fibril. Scale bars are 2 µm.

Subsequently, the influence of doping ratios on the micellar morphologies was investigated. Remarkably, compared to the undoped micelles shown in Figure 2a, the length of fibrils increased gradually after doping (Figure 3a–d). It is noteworthy that as the *r*-value reached 0.4, a large portion of these fibrils underwent end-to-end coupling to form segmented supramolecular micelles, as indicated by the white arrows in Figure 3d. With the further increase in *r*-values, the fibrils started to undergo side-by-side coupling as well (Figure 3e,f), forming nanoribbon-like structures, as indicated by the yellow arrows. With even higher *r*-values, the nanoribbon-like structures further rolled to form nanotube-like structures (Figure 3g,h). With a high doping ratio (*r* = 1), the PHAT4MA block formed superlattices (Figure S15). This very strong LC ordering effect could possibly dictate the self-assembly behaviors. Consequently, the self-assembly process of the one with a lower doping ratio (*r* = 1) was investigated in detail.

The insertion of TNF molecules in between HAT discotic mesogens directly influenced the self-assembly behaviors of P*t*BA_102_-*b*-PHAT4MA_17_ tremendously. The mass block ratio changed from 0.99:1.00 for pristine BCP to 0.91:1.00 (corona:core, w.t.) after doping (*r* = 0.4). However, only the lengths of these rod-like micelles increased significantly, and not their morphologies. This suggests that with the formation of EDA complexes, which could greatly facilitate their alignment in columns within the mesophase, their LC ordering effect was strongly enhanced consequently. Additionally, it is plausible that the CT effect between EDA complexes at the interface between different micelles facilitated the end-to-end coupling. With further increasing doping ratios (*r* ≥ 0.4), the CT effect became sufficiently strong to overcome the steric hindrance provided by the coronal layer to facilitate side-by-side coupling. Although the inter-mesogen spacing was limited by the *van der Waals* radius of carbon [58,59], and could not further decrease after doping (Figure S16), the phase-transition temperature increased to 138 °C (*r* = 0.4, Figure S17), also confirming the boost of LC orderedness after doping.

In addition to the TEM characterizations, the hydrodynamic diameter of the fibrils was also determined with dynamic light scattering (DLS), as shown in Figure S18. However, due to the fibrillar structures and polydispersed length distribution, the hydrodynamic diameter was determined to be 308.7 nm, which is significantly lower than the fibril lengths observed from their TEM image (935.2 nm), and the PDI value was very high.

**Figure 4:**
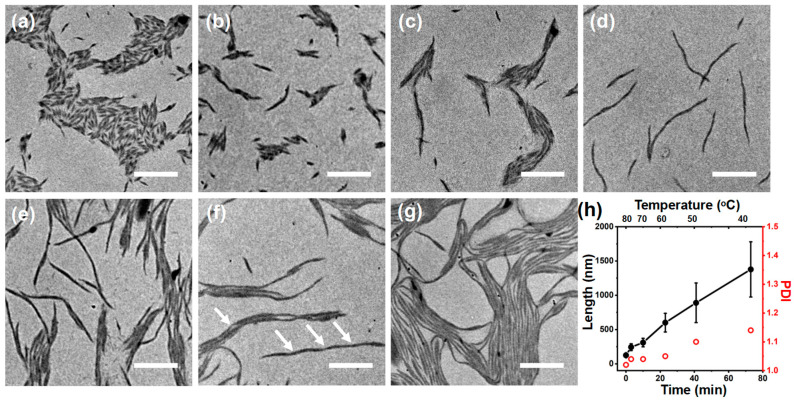
TEM images of self-assembled structures of P*t*BA_102_-*b*-PHAT4MA_17_ (0.5 mg/mL) doped with *r* = 0.4 during the cooling process (80 °C for 1 h and cooling down to r.t. naturally). (**a**) 80 °C, (**b**) 75 °C, (**c**) 70 °C, (**d**) 65 °C, (**e**) 50 °C, (**f**) 40 °C, and (**g**) 21 °C in 2-PrOH solvent. Scale bars are 500 nm. (**h**) Variation of the fibril length and PDI versus time and solution temperatures. The white arrows were indicated to the fibrils end-to-end coupling connection. Error bars represent the mean standard deviation.

To monitor the formation process of fibrils, aliquots were taken from the 2-PrOH solution (*r* = 0.4) at different temperatures during the cooling process and rapidly dried on a TEM grid within a few seconds before observation. As shown in Figure 4a, well-defined short rod-like structures were formed initially at 80 °C, which were quite uniform in length (number average length, *L*_n_ = 124.2 nm, weight average length *L*_w_ = 127.7 nm) with a polydispersity (PDI = *L*_w_/*L*_n_) lower than 1.10. The length of these short rods increased almost linearly with the decreasing temperature (Figure 4b–d) and reached 890 nm at 50 °C (PDI = 1.10) within 41 min (Figure 4e). Subsequently, the lengths of these fibrils were further increased to 1377 nm (PDI = 1.14) after 73 min, when the solution temperature dropped to 40 °C (Figure 4f). When the temperature dropped to r.t. (21 °C), the length became too long and unmeasurable with further cooling. It is noteworthy that not only did the length of these fibrils increase significantly, but their length PDI increased obviously as well. This finding agrees with the observation that these fibrils were grown via both nucleation growth and end-to-end coupling modes.

**Figure 5:**
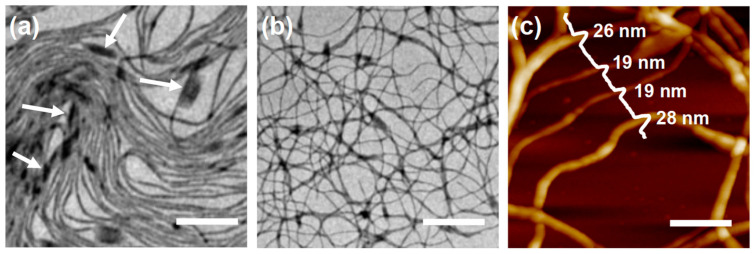
TEM images of the self-assembled structures produced by dispersing the P*t*BA_102_-*b*-PHAT4MA_17_ in 2-PrOH (0.5 mg/mL) at 80 °C for 1 h and cooling down to r.t. naturally, and aging at r.t. for (**a**) 50 days and (**b**) 180 days. (**c**) AFM image of the fibril sample shown in image (**b**). The white arrows were indicated to the formation of nanoribbon-like structures through side-by-side coupling of the individual fibril. Scale bars are 500 nm.

With the further aging of this sample (*r* = 0.4) at r.t., not only were the fibrils elongated, but they were also connected via side-by-side coupling. After 50 days of aging, many fibrils fused into bundle segments, which could be observed in the sample (Figure 5a), as indicated by the white arrows. With further aging (180 days, Figure 5b), network-like structures were formed, in which long fibrils became highly entangled. The fusion of fibrils at the bundle segments was confirmed via atomic force microscopy (AFM) (Figure 5c), which appeared to be much thicker, with a height of approximately 27 nm, than the individual fibrils (~19 nm).

**Figure 6:**
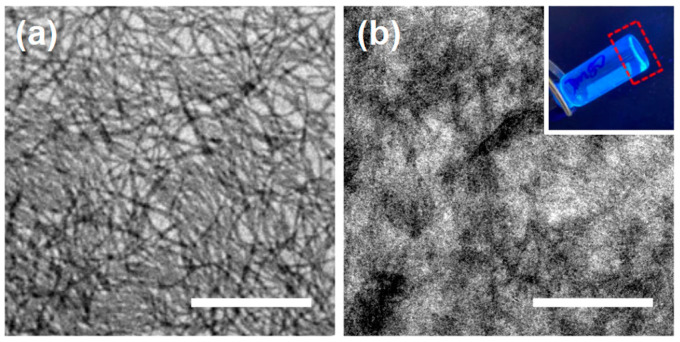
TEM images of the self-assembled structures produced by dispersing the P*t*BA_102_-*b*-PHAT4MA_17_ in 2-PrOH at 80 °C for 1 h and cooling down to r.t. naturally at a concentration of (**a**) 5 mg/mL or (**b**) 10 mg/mL. Scale bars are 1 μm. Inset is a photographic image (irradiated by 365 nm UV light) of the organogel (red dashed square) formed produced by dispersing the P*t*BA_102_-*b*-PHAT4MA_17_ in 2-PrOH at 10 mg/mL.

Since the coupling process was mainly diffusion-controlled for such large nanostructures, higher concentrations could promote the possibility of the fibrils colliding and fusing. With low concentrations (0.1 mg/mL), even long-term aging (r.t. for 50 days) would only result in the elongation of the fibrils instead of the forming of bundles or networks (Figure S19). In order to observe the morphologies of fibrils after aging for a longer period of time, the samples were aged for 360 days at r.t. with different doping ratios (*r* = 0.2, 0.4 and 1.0, respectively). As shown in Figure S20, the morphologies of the fibrillar micelles did not change significantly after aging for 360 days, suggesting that the fibrils had already reached a state of thermodynamic stability and equilibrium after a period of aging. Meanwhile, the formation of networks could be dramatically accelerated by increasing the solution concentration. As shown in Figure 6a, with the BCP concentration increased significnatly to 5.0 mg/mL, a three-dimensional network was formed immediately after the solution was cooled down to r.t. These fibrils were highly entangled, but it was hard to distinguish the contributions from the physical entanglement and bundling of these ultralong fibrils. Moreover, when the concentration was further increased to 10 mg/mL (Figure 6b), the viscosity of the fibrillar solution increased dramatically to form organogel after the solution was cooled down to r.t. (inset in Figure 6b), resulting from the intensively entangled long fibrils. Because of the high density of fibril bundles and intensive entanglement, this gelation’s concentration was much lower than the values (typically higher than 2 wt.%) reported in other BCP organogel systems [60,61,62].

## 4. Summary

In summary, the BCP with an LC block containing HAT discotic mesogen and short methylene side chains was synthesized successfully. The LC orderedness was enhanced significantly by reducing the interference of flexible chains on the interactions between the HAT discotic mesogens, and doping the mesogens with small-molecular electron acceptors. Significantly, this strong LC ordering effect could dictate the self-assembly behaviors of the BCP. Consequently, the BCP could assemble into long fibrils, which grew via both nucleation growth and end-to-end coupling modes. Moreover, with long-term aging, the long fibrils could further couple in a side-by-side manner to form bundles and eventually a three-dimensional network. Meanwhile, by greatly increasing the solution’s concentration, highly entangled fibrils were generated immediately to produce organogel at a decent concentration. We believe this finding broadens the field of controlled assembly methods of LC BCPs, providing new insights into the role of the LC’s ordering effects on the solution-state self-assembly behaviors of BCPs.

## Data Availability

The original contributions presented in the study are included in the article/Supplementary Material, further inquiries can be directed to the corresponding authors.

## Supplementary Materials

The following supporting information can be downloaded at: https://www.mdpi.com/article/10.3390/polym16233339/s1, Figure S1. ^1^H NMR spectrum of the 1,2-dibutoxybenzene. CD_2_Cl_2_ was used as the solvent. Figure S2. ^1^H NMR spectrum of the HAT4. CD_2_Cl_2_ was used as the solvent. Figure S3. ^1^H NMR spectrum of the HAT4-OH. CD_2_Cl_2_ was used as the solvent. Figure S4. ^1^H NMR spectrum of the HAT4-6OH. CD_2_Cl_2_ was used as the solvent. Figure S5. ^1^H NMR spectrum of the HAT4MA. CD_2_Cl_2_ was used as the solvent. Figure S6. ^13^C NMR spectrum of the HAT4MA. CDCl_3_ was used as the solvent. Figure S7. MALDI-TOF-MS spectrum of the HAT4MA. Figure S8. FT-IR spectrum of the HAT4MA. Figure S9. ^1^H NMR spectrum of the TNF. CD_2_Cl_2_ was used as the solvent. Figure S10. ^1^H NMR spectrum of the P*t*BA_102_-Br. CD_2_Cl_2_ was used as the solvent. Figure S11. Synthesis route of P*t*BA_102_-*b*-PHAT4MA_17_. Figure S12. The GPC trace of P*t*BA_102_-Br (black) and P*t*BA_102_-*b*-PHAT4MA_17_ (red). Figure S13. DSC trace of the bulk P*t*BA_102_-*b*-PHAT4MA_17_ block copolymer after annealing. Figure S14. The orthogonal polarized light optical microscopic image (POM) of P*t*BA_102_-*b*-PHAT4MA_17_ upon heating at 21 °C; 126 °C; 128 °C and 130 °C. Scale bars are 300 µm. Figure S15. WAXS spectrum of the structure of the block copolymer doping with TNF (*r* = 1.0). Figure S16. WAXS spectrum of the structure of the block copolymer doping with TNF (*r* = 0.4). Figure S17. DSC trace of the block copolymer doped with TNF (*r* = 0.4). Figure S18. The DLS result of fibrils sample in 2-PrOH at r.t. (0.1 mg/mL, *r* = 0.4). Figure S19. TEM image of the assemblies from the doped P*t*BA_102_-*b*-PHAT4MA_17_ (0.1 mg/mL, *r* = 0.4) in 2-PrOH after aging 50 days at r.t. Figure S20. The TEM image of the self-assembled fibrils sample for aging at r.t. for 360 days. (a) *r* = 0.2, (b) *r* = 0.4, (c) *r* = 1.0. Scale bars are 2 μm. Table S1. Molecular characteristics of P*t*BA-*b*-PHAT4MA.
