# Supplementary material for: Self-Assembly of the Block Copolymer Containing Discotic Mesogens Driven by Liquid Crystalline Ordering Effect
Source: Polymers (Basel). 2024 Nov 28;16(23):3339. doi: 10.3390/polym16233339 (PMC11644099; doi:10.3390/polym16233339)
Supplement: Supplementary file 1 [file polymers-16-03339-s001.zip › polymers-3308788-supplementary.pdf]

Supplementary Materials for

Self-Assembly of the Block Copolymer Containing Discotic Mesogens Driven by Liquid Crystalline Ordering Effect

Xiaojian Hou [1], Lingjuan Hu [1], Huanzhi Yang [1], Bixin Jin [1,2,*], Yunjun Luo [1] and Xiaoyu Li [1,*]

[1] School of Materials Science and Engineering, Key Laboratory of High Energy Density Materials (MOE), Beijing Institute of Technology, Beijing 100081, China
[2] Beijing Institute of Technology, Zhuhai Campus, No. 6 Jinfeng Road, Tangjiawan, Xiangzhou District, Zhuhai 519000, China
* Correspondence: bixinjin@bit.edu.cn (B.J.); xiaoyuli@bit.edu.cn (X.L.)

Synthesis of 1,2-dibutoxybenzene

In a three-neck flask under nitrogen atmosphere, catechol (44.0 g, 399.6 mmol), bromobutanes (219 g, 1598.4 mmol), K_2CO_3 (220.9 g, 1598.4 mmol), KI (6.6g, 40 mmol, catalytic quantity) were stirred in a mixture of acetone and EtOH (1/1, volume ratio) (250 mL) and heated at reflux for 24 h. The organic layer was filtered and the solvent was removed under a vacuum. The crude product was purified *via* column chromatography (silica gel, PE/CH_2Cl_2 = 10:1, volume ratio) to obtain the pale yellow oily product, weighing 85.16 g with 96 % yield. The ^{1}H NMR spectrum is shown in Figure S1.

^{1}H NMR δ_H (ppm) (400 MHz, $CDCl_3$): 6.90 (s, 4H), 4.02-3.99 (t, 4H), 1.84-1.77 (m, 4H), 1.56-1.47 (m, 4H), 1.01-0.97 (t, 6H).

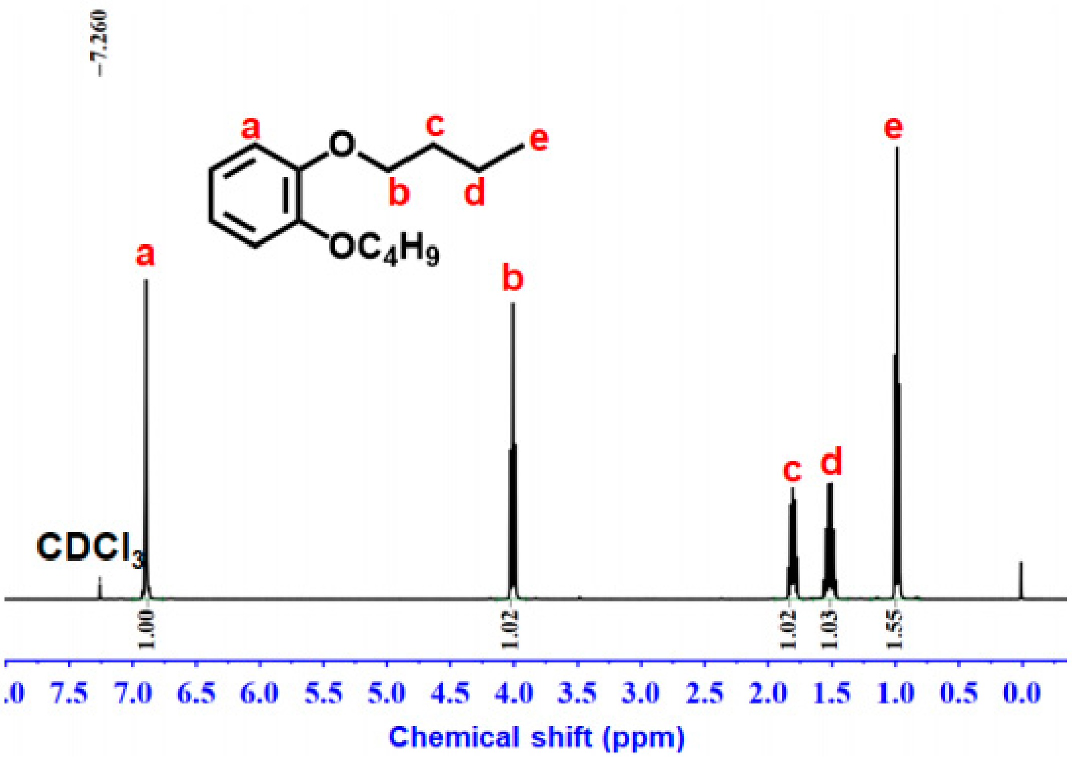

Figure S1. ^{1}H NMR spectrum of the 1,2-dibutoxybenzene. CD_2Cl_2 was used as the solvent.

Synthesis of 2,3,6,7,10,11-hexakis(butoxy)triphenylene (HAT4)

In a three-neck flask under a nitrogen atmosphere, 1,2-dibutoxybenzene (22.22 g, 100 mmol) was added to a vigorously-stirred suspension of iron (III) trichloride (43.33 g, 250 mmol) in anhydrous CH_2Cl_2 (80 mL). The mixture was stirred at r.t. for 3 h with 3 mL H_2SO_4 as the catalyst. The mixture was added to MeOH (500 mL) to quench the reaction. The crude product was then purified by column chromatography and recrystallized from ethanol to yield 14.72 g of white solid with a yield of 73 %. The ^{1}H NMR spectrum is shown in Figure S2.

^{1}H NMR δ_H (ppm) (400 MHz, $CDCl_3$): 7.84 (s, 6H), 4.26-4.23 (t, 12H), 1.97-1.90 (m, 12H), 1.66-1.57 (m, 12H), 1.07-1.03 (s, 18H).

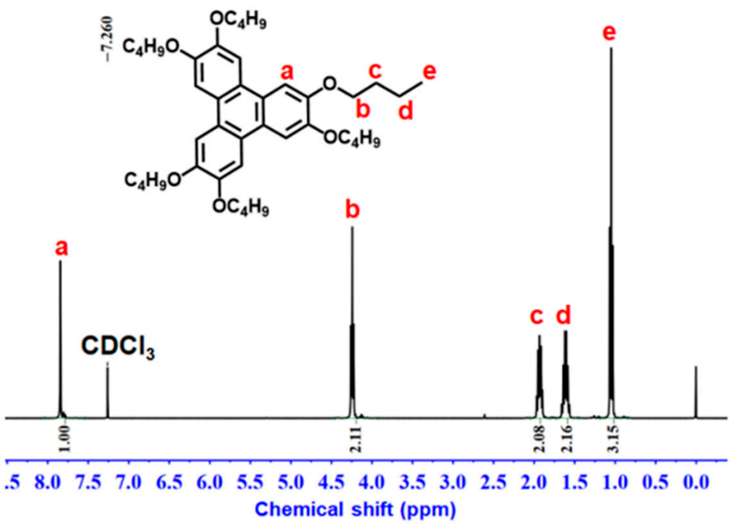

Figure S2. ^{1}H NMR spectrum of the HAT4. CD_2Cl_2 was used as the solvent.

Synthesis of 2- hydroxy-3,6,7,10,11- pentakis(butoxy)triphenylene (HAT4-OH)

A solution of 2,3,6,7,11-hexakis(butoxy)triphenylene (13.2 g, 20 mmol) was dissolved in anhydrous CH_2Cl_2 (140 mL) and cooled to 0 °C. The bromocatecholboronane solution in CH_2Cl_2 (48 mL, 24 mmol) was added under a nitrogen atmosphere and the mixture was stirred at r.t. for 24 h. The mixture was poured over ice water and extracted with CH_2Cl_2. The combined extract was dried with anhydrous Na_2SO_4 overnight. The solvent was removed under a vacuum and the crude product was purified *via* column chromatography (silica gel, PE/CH_2Cl_2 = 4:3, volume ratio) to afford a white solid (5.2 g, 43% yield). The ^{1}H NMR spectrum is shown in Figure S3.

^{1}H NMR δ_H (ppm) (400 MHz, $CDCl_3$): 7.96 (s, 1H), 7.83-7.78 (m, 5H), 5.90 (s, 1H), 4.32-4.20 (m, 10H), 1.96-1.89 (m, 10H), 1.64-1.55 (m, 10H), 1.08-1.03 (m, 15H).

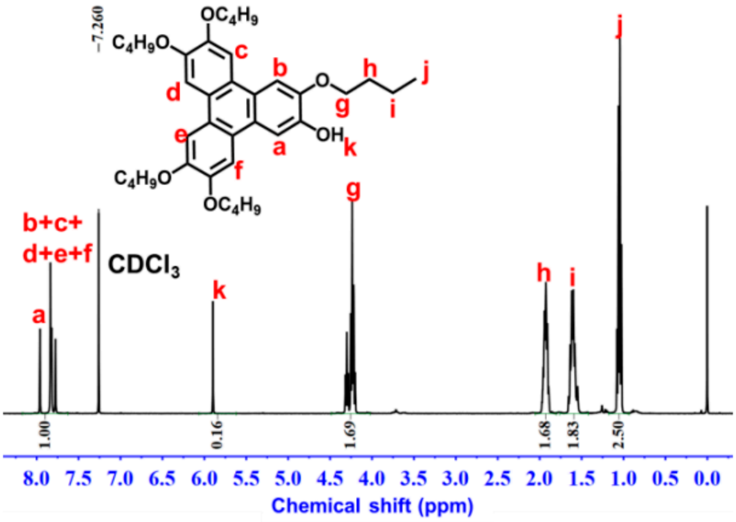

Figure S3. ^{1}H NMR spectrum of the HAT4-OH. CD_2Cl_2 was used as the solvent.

Synthesis of 2- (6-Hydroxyhexoxy) -3,6,7,10,11-pentakis(butoxy) triphenylene (HAT4-6OH)

In a three-neck flask under nitrogen atmosphere, 2-hydroxy-3,6,7,10,11-pentakis(butoxy)triphenylene (1.69 g, 2.8 mmol), 6-bromo-1-hexanol (1.0 g, 5.6 mmol), K_2CO_3 (0.387 g, 5.6 mmol) and KI (0.005 g, 0.3 mmol, catalytic quantity) were stirred in CH_3CN (60 mL) and heated at 80 ^{o}C for 24 h. After cooling to r.t., the organic layer was filtered and the solvent was removed under reduced pressure. The crude product was purified *via* column chromatography (silica gel, PE/CH_2Cl_2 = 1:4, volume ratio) and recrystallized from ethanol to yield 1.66 g of white solid with a yield of 84%. The ^{1}H NMR spectrum is shown in Figure S4.

^{1}H NMR δ_H (ppm) (400 MHz, $CDCl_3$): 7.84 (s, 6H), 4.26-4.23 (t, 12H), 3.71-3.67 (m, 2H), 1.98-1.90 (m, 12H), 1.67-1.41 (m, 16H), 1.07-1.03 (t, 15H).

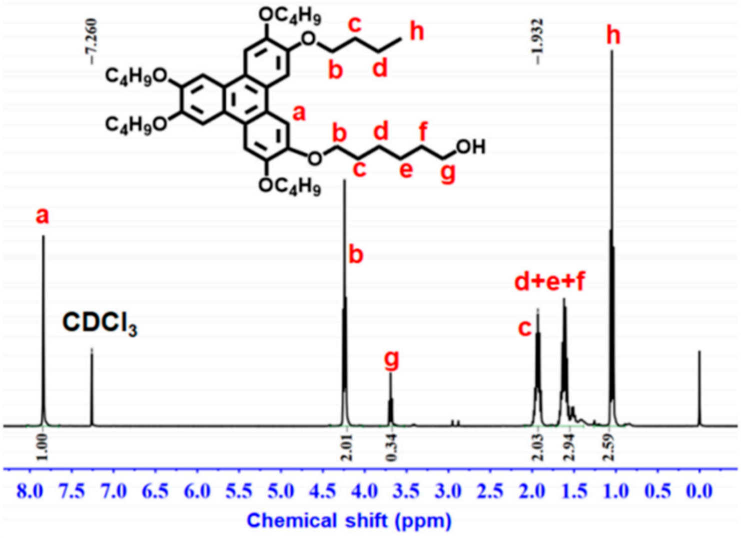

Figure S4. ^{1}H NMR spectrum of the HAT4-6OH. CD_2Cl_2 was used as the solvent.

Synthesis of 2- (6-methylacrylate) hexoxy-3,6,7,10,11-pentakis(butoxy) triphenylene (HAT4MA)

In a three-neck flask under a nitrogen atmosphere, 2- (6-Hydroxyhexoxy) -3,6,7,11-pentakis(butoxy)triphenylene (0.9 g, 1.06 mmol) and TEA (0.33 mL, 2.34 mmol) were dissolved in dry CH_2Cl_2 (20 mL) and cooled to 0 ^{o}C. Methacryoyl chloride (0.2 mL, 2.12 mmol) was added dropwise to the reaction mixture. The solution was allowed to warm up to r.t. and stirred for 10 h. After evaporation of the solvent, the crude product was purified *via* column chromatography (silica gel, PE/CH_2Cl_2 = 4:1, volume ratio) to yield 0.86 g of white solid with a yield of 89 %. The ^{1}H NMR, ^{13}C NMR, MALDI-TOF-MS spectra, and FT-IR spectra are shown in Figures S5, S6, S7, and S8, respectively.

^{1}H NMR δ_H (ppm) (400 MHz, $CDCl_3$): 7.84 (s, 6H), 6.10 (s, 1H), 5.54-5.53 (t, 1H), 4.26-4.17 (m, 14H), 1.98-1.89 (m, 15H), 1.66-1.56 (m, 16H), 1.06-1.03 (m, 15H);

^{13}C NMR δ_H (ppm) (101 MHz, $CDCl_3$): 167.69, 149.14, 149.01, 136.65, 125.36, 123.79, 123.75, 123.73, 107.50, 69.84, 69.72, 69.54, 64.80, 31.63, 29.53, 28.80, 26.03, 26.01, 19.51, 18.47, 14.11;
FT-IR (cm^{-1}): 837.69, 1048.23, 1168.64, 1256.92, 1386.26, 1435.03, 1515.96, 1616.80,1718.92, 2856.10, 2925.34;

MALDI-TOF-MS: calcd. for $C_{48}H_{68}O_8$ 773.06, found 772.4; EA:calcd. for $C_{48}H_{68}O_8$ C, 74.58; H, 8.87; O, 16.56; found: C, 74.59; H, 8.92; O, 16.49.^{1}H NMR δ_H (ppm) (400 MHz, $CDCl_3$): 7.84 (s,

6H), 4.26-4.23 (t, 12H), 3.71-3.67 (m, 2H), 1.98-1.90 (m, 12H), 1.67-1.41 (m, 16H), 1.07-1.03 (t, 15H).

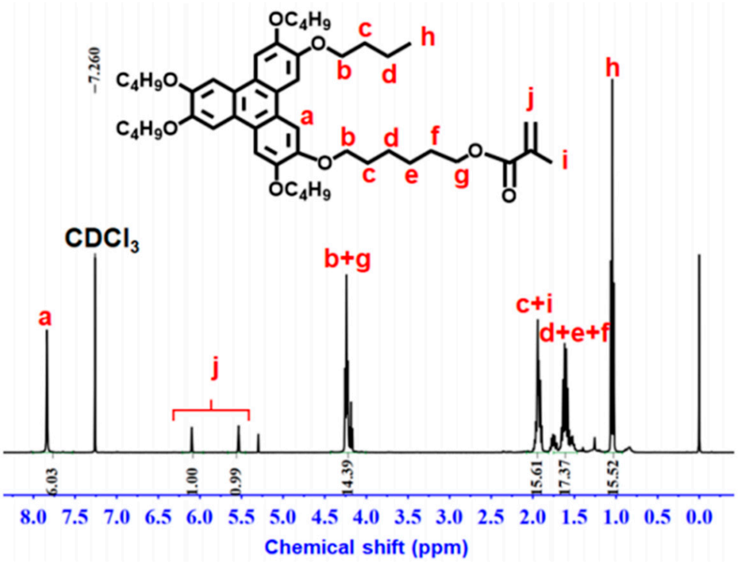

Figure S5. ^{1}H NMR spectrum of the HAT4MA. CD_2Cl_2 was used as the solvent.

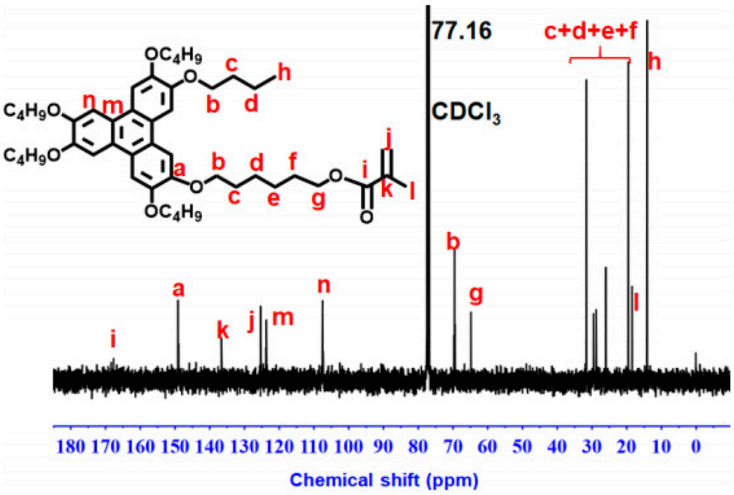

Figure S6. ^{13}C NMR spectrum of the HAT4MA. $CDCl_3$ was used as the solvent.

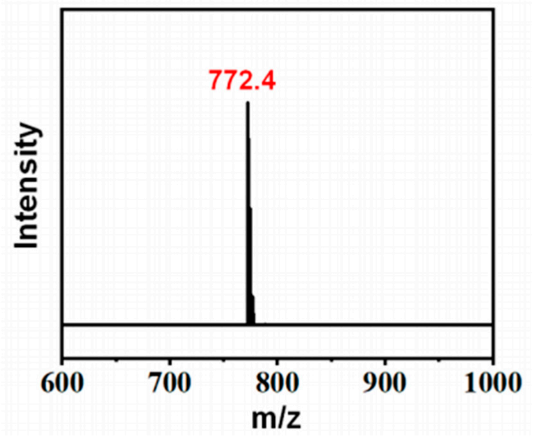

Figure S7. MALDI-TOF-MS spectrum of the HAT4MA.

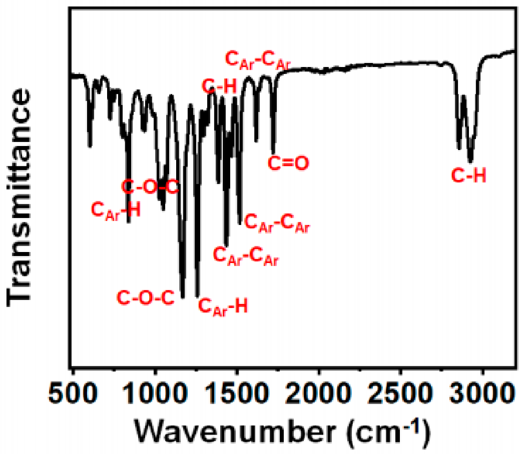

Figure S8. FT-IR spectrum of the HAT4MA.

Synthesis of 2,4,7-trinitro-9H-fluoreno-9-ketone (TNF)

Into a stirred solution of HNO_3 (15.8 M, 40 mL) and H_2SO_4 (18 M, 40 mL), 9-fluorenone (1.80 g, 0.01 mol) was added at r.t. and heated to reflux for 2 h (105 °C, internal). The solution was allowed to warm up to r.t. and the reaction mixture was then poured into 200 mL ice water. The precipitated powder was collected by suction filtration, washed with water, and dried. The yellow solid was further purified *via* repeated precipitation from CH_2Cl_2 solution into MeOH, and dried under reduced pressure. The resulting yellow solid was dried under vacuum at r.t. to yield 2.6 g of yellow solid with 82 % yield. The ^{1}H NMR spectrum is shown in Figure S9.
^{1}H NMR δ_H (ppm) (400 MHz, $CDCl_3$): 9.03-9.02 (d, 1H), 8.83-8.82 (d, 1H), 8.68-8.67(d, 1H), 8.59-8.56 (m, 1H), 8.39-8.36 (d, 1H).

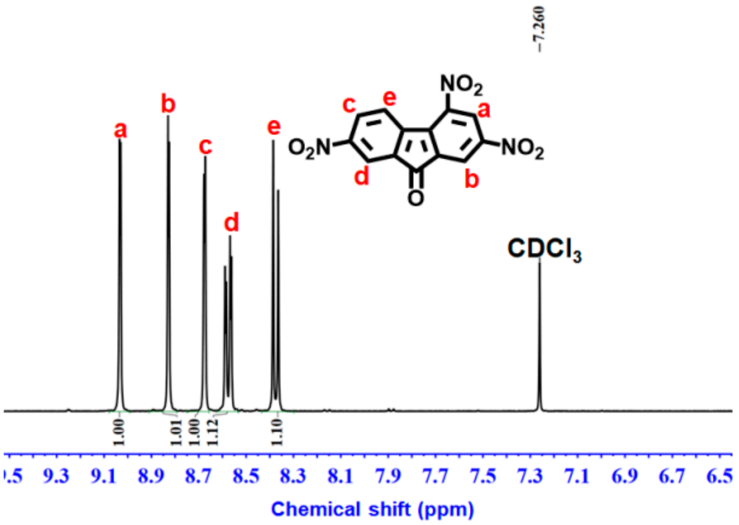

Figure S9. ^{1}H NMR spectrum of the TNF. CD_2Cl_2 was used as the solvent.

Synthesis of P*t*BA$_{102}$-Br

The P*t*BA$_{102}$-Br were synthesized *via* ATRP with CuBr (36.0 mg, 0.25 mmol), PMDETA (52.5 μL, 0.25 mmol), *t*BA (5.44 mL, 37.5 mmol), HEBiB (38.25 μL, 0.25 mmol) and 3 mL toluene were introduced into a Schlenk tube and degassed with three freeze-pump-thaw cycles. Subsequently, the polymerization solution was heated at 60 °C for 6 h under a nitrogen atmosphere with vigorous stirring. Cupreous salt was removed by filtering the reaction solution through Al_2O_3 columns, and the polymer was further purified *via* repeated precipitations from THF solution into a mixture of water and methanol (7/3, volume ratio), and

dried under reduced pressure. A white solid of P*t*BA was obtained (2.19 g, yield 45 %). The ^{1}H NMR characterization results, shown in Figure S10, indicate a characteristic peak at 2.22 ppm for P*t*BA$_{102}$, confirming the successful synthesis of the final polymer with the defined chemical structure by ATRP.

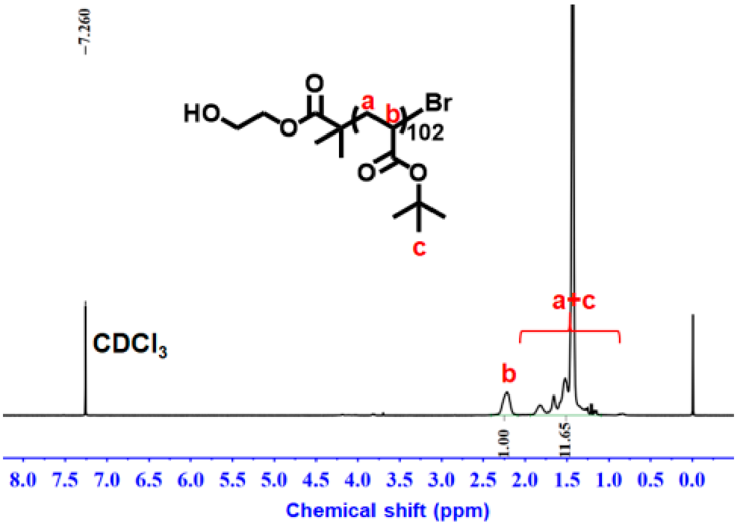

Figure S10. ^{1}H NMR spectrum of the P*t*BA$_{102}$-Br. CD_2Cl_2 was used as the solvent.

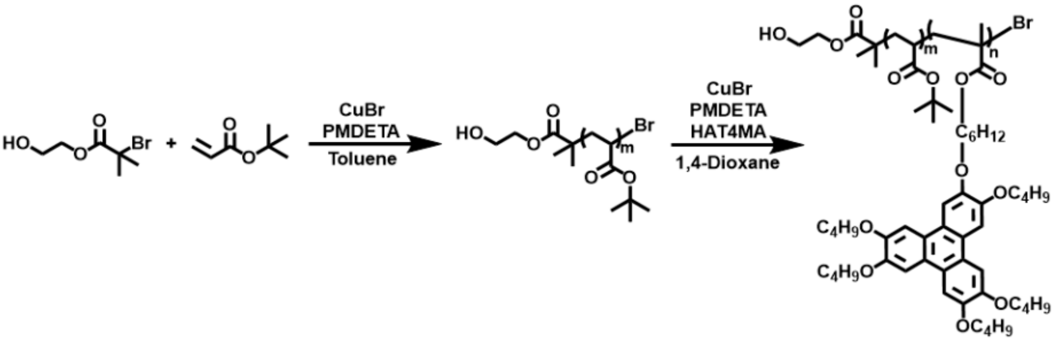

Figure S11. Synthesis route of P*t*BA$_{102}$-*b*-PHAT4MA$_{17}$.

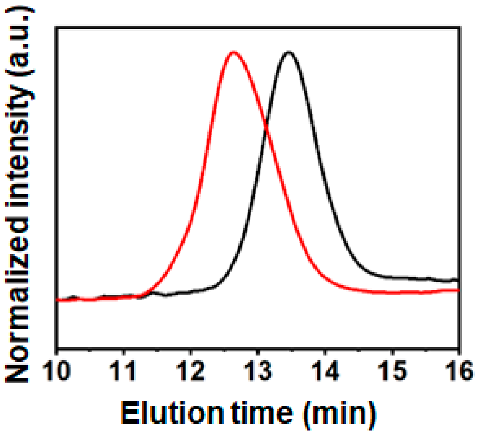

Figure S12. The GPC trace of P*t*BA$_{102}$-Br (black) and P*t*BA$_{102}$-*b*-PHAT4MA$_{17}$ (red).

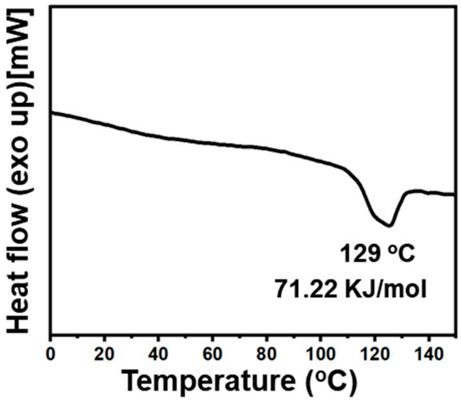

Figure S13. DSC trace of the bulk P*t*BA$_{102}$-*b*-PHAT4MA$_{17}$ block copolymer after annealing.

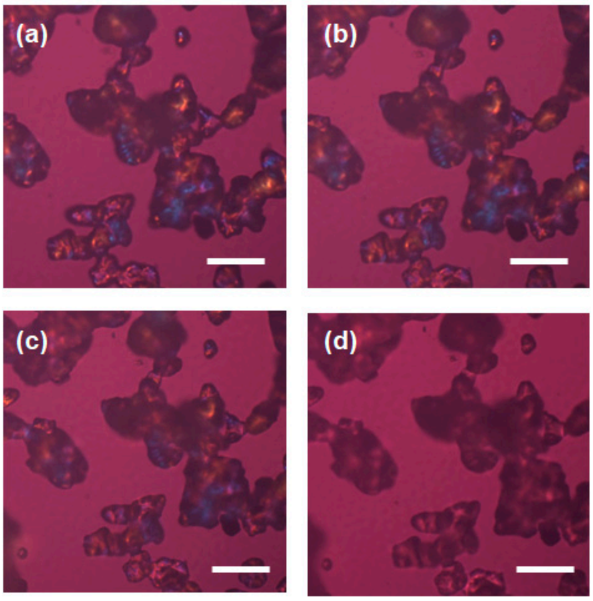

Figure S14. The orthogonal polarized light optical microscopic image (POM) of P*t*BA$_{102}$-*b*-PHAT4MA$_{17}$ at 21 °C; 126 °C; 128 °C and 130 °C. Scale bars are 300 μm.

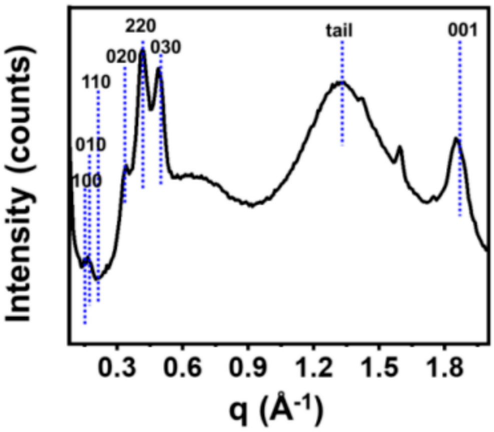

Figure S15. WAXS spectrum of the structure of the block copolymer doping with TNF (r = 1.0).

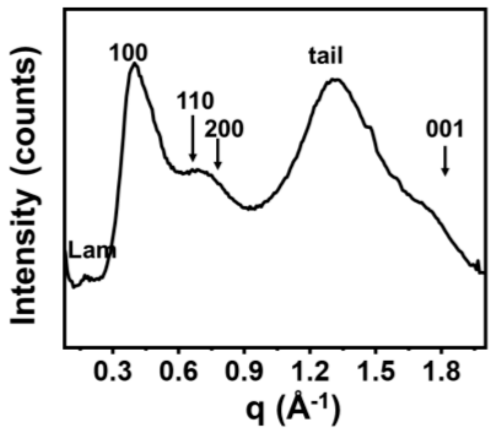

Figure S16. WAXS spectrum of the structure of the block copolymer doping with TNF (r = 0.4).

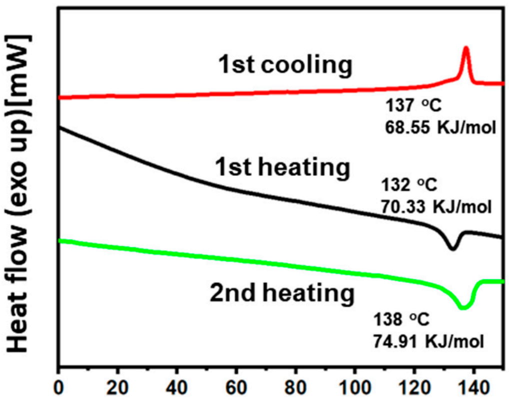

Figure S17. DSC trace of the block copolymer doped with TNF (r = 0.4).

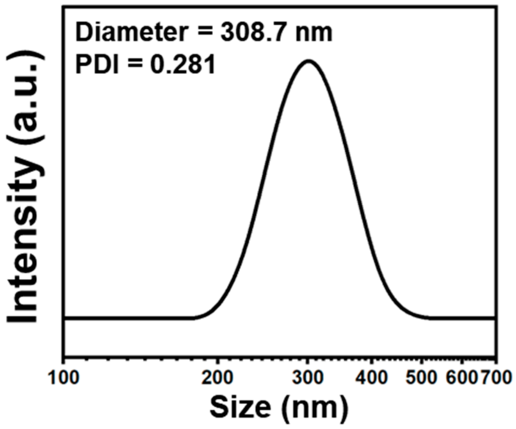

Figure S18. The DLS result of fibrils sample in 2-PrOH at r.t. (0.1 mg/mL, r = 0.4).

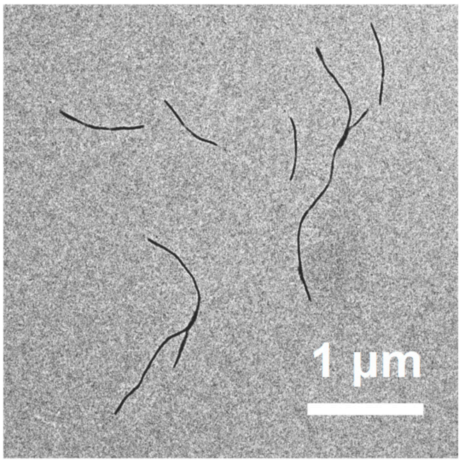

Figure S19. TEM image of the assemblies from the doped P*t*BA$_{102}$-*b*-PHAT4MA$_{17}$ (0.1 mg/mL, r = 0.4) in 2-PrOH after aging 50 days at r.t.

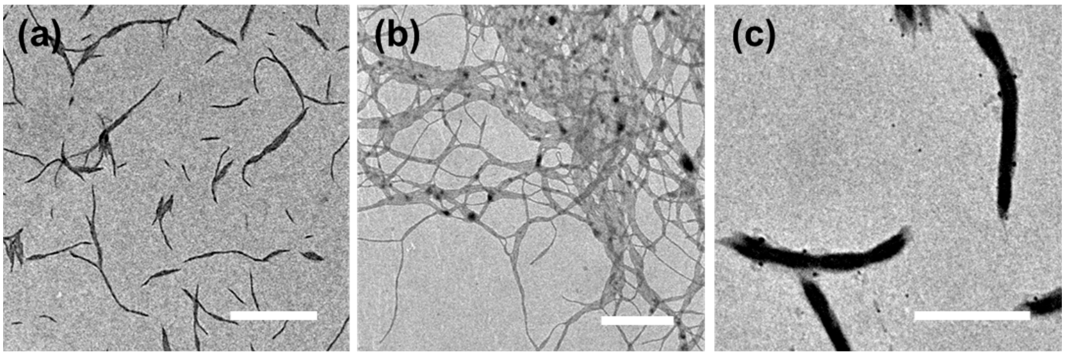

Figure S20. The TEM image of the self-assembled fibrils sample for aging at r.t. for 360 days. (a) r = 0.2, (b) r = 0.4, (c) r = 1.0. Scale bars are 2 μm.

Table S1. Molecular characteristics of P*t*BA-*b*-PHAT4MA.

Polymer	M_n (g/mol)[a]	M_w (g/mol)[a]	M_w/M_n[a]	m[b]	n[b]
P*t*BA-Br	13300	15000	1.13	102	-
P*t*BA-*b*-PHAT4MA	21100	26200	1.24	102	17

[a] : obtained from GPC; [b] : calculated from the value of m and the results from ^{1}H NMR;
m and *n* are the degrees of polymerization of the corona and core forming block, respectively.
